# Mesothelin-Targeted Recombinant Immunotoxins for Solid Tumors

**DOI:** 10.3390/biom10070973

**Published:** 2020-06-28

**Authors:** Brendan L. Hagerty, Guillaume J. Pegna, Jian Xu, Chin-Hsien Tai, Christine Alewine

**Affiliations:** 1 Laboratory of Molecular Biology, Center for Cancer Research, NCI, NIH, Bethesda, MD 20892, USA; brendan.hagerty@nih.gov (B.L.H.); will.pegna@nih.gov (G.J.P.); jian.xu@nih.gov (J.X.); taic@mail.nih.gov (C.-H.T.); 2 Surgical Oncology Program, Center for Cancer Research, NCI, NIH, Bethesda, MD 20892, USA; 3 Medical Oncology Service, Center for Cancer Research, NCI, NIH, Bethesda, MD 20892, USA

**Keywords:** immunotoxin, mesothelin, mesothelioma, pancreatic adenocarcinoma

## Abstract

Mesothelin (MSLN) is a cell surface glycoprotein normally expressed only on serosal surfaces, and not found in the parenchyma of vital organs. Many solid tumors also express MSLN, including mesothelioma and pancreatic adenocarcinoma. Due to this favorable expression profile, MSLN represents a viable target for directed anti-neoplastic therapies, such as recombinant immunotoxins (iToxs). Pre-clinical testing of MSLN-targeted iTox’s has yielded a strong body of evidence for activity against a number of solid tumors. This has led to multiple clinical trials, testing the safety and efficacy of the clinical leads SS1P and LMB-100. While promising clinical results have been observed, neutralizing anti-drug antibody (ADA) formation presents a major challenge to overcome in the therapeutic development process. Additionally, on-target, off-tumor toxicity from serositis and non-specific capillary leak syndrome (CLS) also limits the dose, and therefore, impact anti-tumor activity. This review summarizes existing pre-clinical and clinical data on MSLN-targeted iTox’s. In addition, we address the potential future directions of research to enhance the activity of these anti-tumor agents.

## 1. Introduction

Mesothelin (MSLN) is a cell surface glycoprotein normally expressed on serosal tissues such as pleura, pericardium, and peritoneum, but is not in the parenchyma of any vital organs [1,2]. It is commonly expressed on a number of solid tumors, such as mesothelioma, pancreatic adenocarcinoma, ovarian cancer and others [1,3,4,5,6,7,8,9]. It has no known physiologic function, but may play a role in tumorigenesis and malignancy [10,11,12,13,14,15]. Because of its strong differential expression, it has become a popular target for a directed anti-neoplastic therapies, including monoclonal antibodies, antibody-drug conjugates (ADCs), radioimmunotherapy (RIT), CAR-T cells, and vaccines [16,17,18,19,20,21].

**Figure 1:**
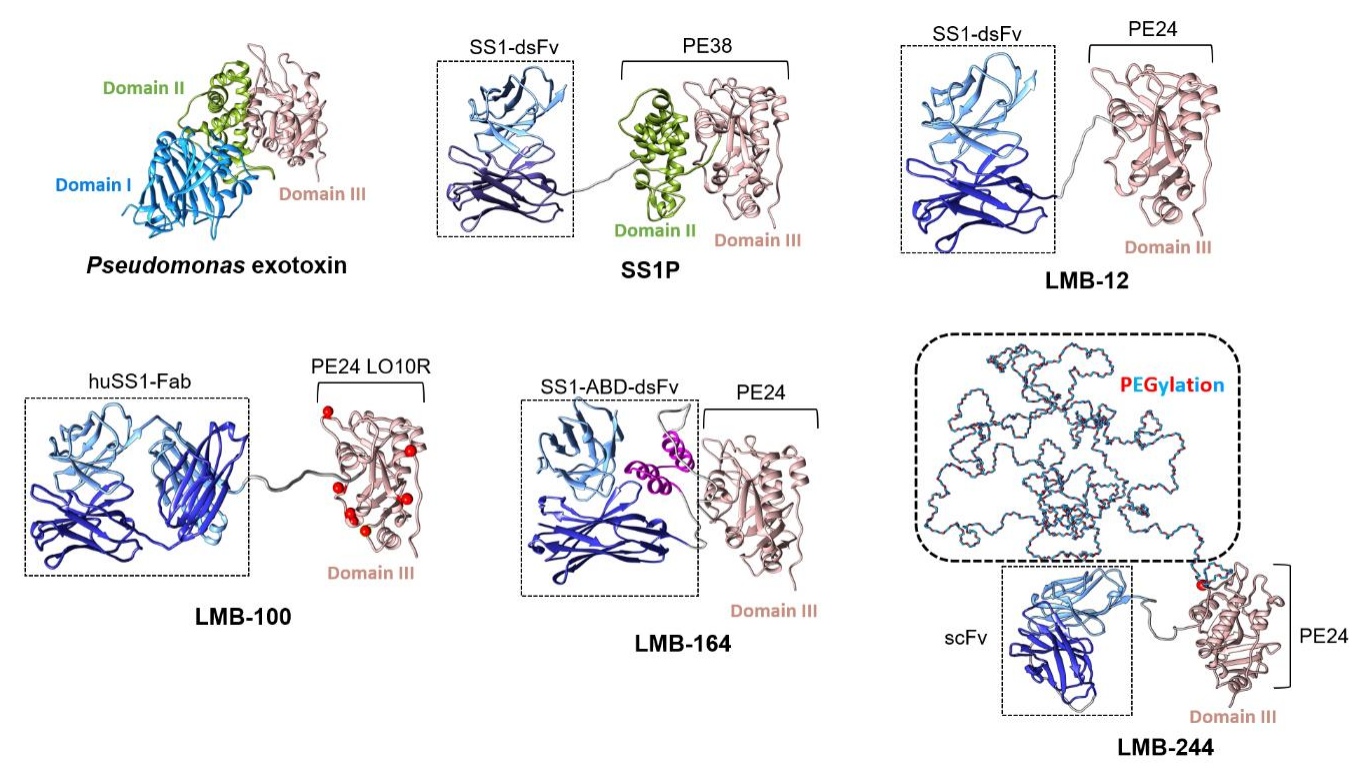
Structures of mesothelin (MSLN)-targeted recombinant immunotoxins (iToxs). Pseudomonas exotoxin (PE) contains three domains: domain I (binding), domain II (linker), and domain III (catalytic). SS1P was engineered with a MSLN-targeted dsFv (SS1) fused to PE38, containing domains II and III of PE. LMB-12 was formed by attaching SS1 to PE24 (only furin cleavage site of domain II remains from PE38), in an effort to eliminate T cell epitopes. LMB-100 contains a humanized anti-MSLN Fab linked to a modified PE24, designed to eliminate remaining B cell epitopes. The red balls in the model indicate individual residues that were mutated during the technical deimmunization. LMB-164 is a derivative of LMB-12, with insertion of an albumin binding domain, shown in lavender. Finally, LMB-244 consists of a single chain Fv (scFv), linked to PE24 that contains a cysteine site-specific PEGylation on the PE24 molecule. The PE38 structure is the X-ray crystallograph of wild type PE structure (PDB:1IKQ). All other iToxs are modeled from the crystal structure of mesothelin and antibody complex (PDB: 4F3F) with the PE38. LMB-164 includes the albumin binding domain modeled from the one in Streptococci (PDB: 1GJS). The domain III of PE with substrate NAD and AMP (PDB: 1DMA) and the complex structure of PE and Elongation factor 2 (PDB: 1ZM4) were superposed to iToxs models, to avoid potential binding interference when generating the LMB-164 and LMB-244 models. Molecular graphics generated with UCSF Chimera were developed by the Resource for Biocomputing, Visualization, and Informatics at the University of California, San Francisco, with support from NIH P41-GM103311.

Recombinant immunotoxins (iTox) are potent cytotoxic molecules consisting of an antibody (or fragment) linked to a plant or bacterial toxin [22]. A number of toxins have been used as payloads, including: ricin [23] diptheria toxin [24] *Pseudomonas* exotoxin A (PE), gelonin [25], and ribotoxins such as α-sarcin [26]. They have undergone testing as treatments for various solid and hematologic malignancies for several decades. The activity of iTox therapy against solid tumors was first reported in 1996, when LMB-1, a (PE)-based immunotoxin targeting a Lewis-y antigen, was used to treat 38 patients with a variety of advanced adenocarcinomas [27]. PE is a highly toxic cellular toxin that catalyzes the irreversible ADP ribosylation of elongation factor-2 (EF-2). This modification inactivates EF-2, a critical and non-redundant enzyme required for protein translation, resulting in a typically fatal inhibition of new protein synthesis in the affected cell. The native PE toxin consists of three domains: a binding domain (I), a linker domain (II), and a catalytic domain (III) (Figure 1).

In iTox, the native binding domain is replaced with a novel targeting molecule, such as anti-Lewis-y antibody, to specifically direct the poison to cancer cells. Truncated PE is inactive outside of the cell, but highly lethal if even a few molecules reach the cytosol, making precise targeting extremely important. 

Pancreatic adenocarcinoma (PDAC) is a lethal disease, with a five-year overall survival of just 10% [28]. Resection with systemic therapy is the only chance for cure when feasible. However, more than 50% of patients present with metastatic disease, rendering them unable to benefit from surgery [28]. Systemic therapy for such patients has limited efficacy, with response rates ranging between 6 and 30% [29,30]. Mesothelioma is also an aggressive solid tumor resistant to systemic treatment [31]. Similar to PDAC, metastatic disease virtually eliminates any reasonable hope for cure. In patients with unresectable diseases, response rates to best medical therapy are modest, with only 40% achieving an objective response [32,33]. Targeted therapies that have shown benefit in other solid tumors, such as immune checkpoint inhibitors or EGFR antagonists, play a limited role in the treatment of PDAC and mesothelioma [34,35,36].

While Lewis-y targeting proved clinically intractable due to unacceptable toxicity in patients, the development of immunotoxins with PE-based payloads and alternative binding domains has continued. Choudhary and colleagues from the laboratory of Ira Pastan reported the synthesis of the first PE-based MSLN-targeted immunotoxin in 1998, and demonstrated anti-tumor activity in mice bearing human tumors expressing MSLN [37]. Since then, MSLN-targeted immunotoxins have been extensively investigated in both clinical and pre-clinical settings, as anti-neoplastic therapies for solid malignancies. Here, we review existing data on those drugs and explore strategies for harnessing and enhancing their activity. 

## 2. Mesothelin as a Target

MSLN is a 40-kDa glycosylated protein found on the cell surface [1]. It was discovered by the Pastan lab in 1992, as an antigen to the K1 antibody isolated from mice bearing human mesothelioma and ovarian tumors [38,39]. It is produced from a 69-kDa precursor protein comprised of the mature MSLN and megakaryocyte potentiating factor (MPF). MPF is cleaved from the precursor protein, then secreted into the extracellular space [2]. Mature membrane-linked MSLN can be shed from the cell surface, and is often measurable in the serum of patients harboring tumors that express it [8,40,41]. Its function on the cellular and molecular level is still unclear, and it has one known binding partner, MUC-16 [14,42,43].

While MSLN does not appear to be a primary driver of malignancy, it has been associated with invasiveness and poor outcomes in multiple malignancies [5,9,44,45,46,47]. There are a variety of mechanisms through which MSLN may act to produce such findings. It is likely that the binding of MUC16 to MSLN promotes cell adhesion to serosal surfaces and may play a role in peritoneal and pleural dissemination [14,42,43]. It has also been shown that MSLN expression induces signaling which renders the cell resistant to anoikis [48]. Furthermore, Bharadwaj et al. demonstrated that MSLN can act through NF-ĸB to produce an autocrine IL-6 signal, which enhances cell survival in human pancreatic cancer cells [49]. Recent evidence has shown that MSLN promotes peritoneal metastases via induction of angiogenesis in the local microenvironment, though the exact mechanism by which this occurs has yet to be elucidated [11]. Despite this apparent functional role in tumors, blockade treatment with a monoclonal antibody had limited effect [19].

MSLN expression is observed in multiple solid malignancies. It is found on virtually all epithelioid mesothelioma and pancreatic cancers [6,50]. It is also frequently seen on the immunostaining of serous, endometrioid and clear cell ovarian cancers, gastric cancers, and non-small cell lung cancers [6]. Because iToxs have a mechanism of action distinct among cancer therapeutics—the disruption of protein synthesis—it is imperative that they are not directed to vital tissues. Although MSLN expression can be induced in fibroblasts during wound healing [51], under normal conditions, MSLN expression is restricted to serosal surfaces, a tissue type that is not critical to organism survival [10]. As a result, MSLN can be effectively targeted by potent cytotoxic agents without significant deleterious effects to the patient. Because of its absence on tissues vital to physiologic homeostasis and a high prevalence in many solid tumors, MSLN presents an good target for iTox therapy. 

## 3. Pre-Clinical Development Pipeline 

All MSLN-targeted iTox reported in the literature carry a PE payload. The laboratory of Ira Pastan has pioneered the development of these MSLN-targeted iToxs for more than two decades. Strong anti-tumor activity of the MSLN-targeted iTox K1-LysPE38QQR was first reported in the pre-clinical models of MSLN-expressing human ovarian cancer and malignant mesothelioma in 2000 [52]. Subsequently, a new iTox called SS1P (SS1dsFv-PE38) was developed by genetic engineering to have superior stability and higher binding affinity than K1-LysPE38QQR [52,53] (Figure 1). SS1P is composed of an anti-MSLN disulphide stabilized Fv (dsFv) and a truncated form of *Pseudomonas* exotoxin, PE38, in which the binding domain (domain I) is deleted and replaced with the dsFv [53]. SS1P demonstrated high cytotoxicity to MSLN-expressing cells. Observed IC_50_’s in vitro against tumor cells obtained from patients with peritoneal mesothelioma ranged from 0.08–3.9 ng/mL [54]. Cell sensitivity was reduced with high MSLN shedding, suggesting that activity was specific for MSLN-expressing cells [41]. Tumor regressions were seen when SS1P was given as monotherapy to nude mice harboring human tumors expressing MSLN [37]. In addition, combination with cytotoxic chemotherapy, such as paclitaxel and gemcitabine, synergistically enhanced the anti-tumor activity of SS1P in tumor-bearing mice [55,56]. 

Despite success in the pre-clinical setting, anti-drug antibody (ADA) formation limited the clinical use of SS1P. Generally, ADAs are produced and secreted by activated B cells through T cell dependent or independent pathways. For the T cell dependent pathway, a protein drug is taken up by antigen presenting cells (APCs) and proteolytically degraded into small peptides. Some of these peptides will specifically interact with human leukocyte antigen (HLA)/major histocompatibility complex (MHC) class II molecules and present on the surface of APCs. Then, the antigen (peptide epitopes)-MHC class II complex is recognized by T helper cells (T_H_ cells). This causes specific activation in the setting of a costimulatory signal generated by APCs, thereby inducing the activation of drug-specific B-cells. For the T cell independent pathway, protein drug-derived epitopes can directly activate B-cells and produce ADA through the classical immune pathway [57,58]. Based on the above, the elimination of B-cell or T cell epitopes has been broadly studied to deimmunize protein-based therapeutics [59,60,61,62]. A less immunogenic successor to SS1P was sought, in which T and B cell epitopes were eliminated from the toxin moiety [63,64,65]. To identify B cell epitopes, regions within PE38 that were reactive with phage display libraries assembled from the F_v_’s of patients who had developed neutralizing ADAs to PE38 iTox were catalogued [65]. T cell epitopes were identified by incubating donor peripheral blood mononuclear cells with PE38 iTox, to simulate exposure to iTox, and then exposing the induced T cells from these samples to overlapping 15-mer peptides occurring in PE38. Reactivity was then measured by T cell IL-2 response [66,67]. Once epitopes were identified, deletions or point mutations were engineered within PE38 that could eliminate these epitopes without significantly reducing activity. This technical deimmunization resulted in multiple iterations of MSLN-targeted iToxs that retained or even enhanced cytotoxicity and possessed decreased immunogenicity [68]. This series utilized a newly designed PE base fragment called PE24. PE24 is 14 kD smaller than PE38, because it lacks the majority of domain II. This region of the molecule contains the most immunogenic T cell epitopes within PE [66]. The insertion of a flexible GGS linker between the canonical furin cleavage site (the preserved region of domain II) and the MSLN binding domain was required to preserve anti-tumor activity in most cancer cell lines [69] (Figure 1). Interestingly, all PE24-based MSLN-targeted iToxs can be safely administered to mice at doses >6-fold higher than SS1P. Furthermore, high doses of the prototype PE24-based iTox SS1-LR (later called LMB-12) could be administered to rats without causing pulmonary edema, a rodent correlate for capillary leak syndrome (CLS) [69].

LMB-100 (formerly RG7787 and RO6927005), a PE24-based iTox, became the clinical lead of this series. This iTox was co-developed by the Pastan lab and F. Hoffmann-La Roche Ltd. (Basel, Switzerland). It consists of a fully humanized Fab, to eliminate the possibility of ADA formation against the murine-derived SS1 F_v_, attached to a modified version of PE24 (PE24LO10R). PE24LO10R includes the large domain II deletion present in PE24 and point mutations at each of the six B-cell epitopes within the toxin catalytic domain [64] (Figure 1). These mutations include: R490A (B cell epitope), R427A (B and T cell shared epitope), R505A (B and T cell shared epitope), R467A (B cell epitope), D463A (B cell epitope), R538A (B cell epitope), and R456A (B cell epitope) [68,70]. LMB-100 has high cytotoxicity on MSLN-expressing cell lines in vitro with IC_50_’s in the range of SS1P [71,72]. In immunodeficient mouse models, LMB-100 treatment slows the growth of MSLN-expressing tumors when used alone [72]. When combined with cytotoxic chemotherapy, tumor regressions were seen in mesothelioma xenograft models [71]. Kollmorgen et al. found that the combination of LMB-100 and cisplatin or paclitaxel showed striking tumor growth inhibition in a PDX model of ovarian cancer as well [73]. Furthermore, synergistic activity was observed with taxanes, such that the combination produced complete tumor responses in a mouse model of pancreatic cancer [74]. 

A growing body of literature supports the investigation of MSLN-targeted iToxs in combination with newer, targeted anti-tumor drugs. For instance, the activated protein kinase C (PKC) inhibitor enzastaurin significantly enhanced the cytotoxicity of SS1P in resistant cells [75]. Leshem et al. reported that SS1P induced immunogenic cell death and increased the anti-tumor efficacy of anti-CTLA-4 based therapy in AE17M mouse mesothelioma tumors [76]. Additionally, the combination of LMB-100 and the discoidin domain receptor 1 (DDR1) inhibitor caused greater growth inhibition in pancreatic cancer KLM1 xenograft tumor models, by decreasing ribosomal protein expression and enhancing the inhibition of protein synthesis [77]. Liu et al. found that the transcription inhibitor Actinomycin D synergistically enhanced the cytotoxicity of LMB-100 on a range of MSLN-expressing cells in vitro, via the upregulation of apoptotic pathways, and caused significant tumor growth inhibition in mouse models [78]. Subsequently, Liu and colleagues also found that the histone deacetylase inhibitor panobinostat synergistically enhanced the cytotoxicity of LMB-100 in numerous cancer cell lines [79]. Notably, other data showed that combination with the pan-JAK inhibitor tofacitinib not only reduced anti-SS1P antibodies in mice, but also increased the in vivo antitumor efficacy of LMB-100 against pancreatic cancer and triple negative breast cancer mouse models [80,81]. The results of these in vitro and in vivo studies indicate that MSLN-targeted iToxs have the potential for clinical anti-tumor activity if optimal combination strategies can be identified.

To further enhance anti-tumor activity and increase biomolecule penetration into high stroma tumors like PDAC, designing iToxs with prolonged serum half-life was pursued. PEGylation is one common strategy used by therapeutic protein engineers to increase half-life. Consistent with this, Filpula et al. reported that PEGylated SS1P showed stronger cytotoxicity on cultured MSLN-overexpressing A431-K5 cells and exhibited higher anti-tumor activity in mouse A431-K5 xenografts [82]. More recently, Zheng et al. reported a 10- to 30-fold increase in the half-life of MSLN-targeted iTox after site-specific PEGylation, and demonstrated the improved anti-tumor activity of these biomolecules in mouse tumor models [83]. An alternative strategy to PEGylation was simultaneously pursued. Wei et al. engineered a series of recombinant MSLN-targeted iTox, bearing an albumin-binding domain between the anti-MSLN F_v_ and the furin cleavage site in Domain II of the toxin (Figure 1). This modification increased serum half-life in mice by more than 10-fold, and greatly enhanced anti-tumor efficacy against a mouse model of pancreas cancer when administered at equal mg dose as LMB-12, previously the most active iTox against this tumor cell type [84]. Further studies in appropriate models will be required, to determine whether increased toxicity accompanies the improved anti-tumor profile of these prolonged half-life iToxs.

## 4. Clinical Experience

PDAC and mesothelioma are lethal malignancies largely refractory to standard therapies. As such, iTox therapy has been pursued as a potential option for patients with these devastating diagnoses. A summary of clinical trials testing MSLN-targeted iToxs is provided in Table 1.

### 4.1. SS1P in the Clinic

SS1P was introduced into the clinic in a phase I trial designed to evaluate safety. The monotherapy was administered as a short infusion to patients with advanced, pre-treated mesothelioma, ovarian, or pancreatic cancer. A maximum tolerated dose (MTD) of 45mcg/kg was established when given every other day for three doses. Minor tumor responses were seen, and 19 of 33 evaluable patients had a stable disease (SD) [85]. Shortly thereafter, a second phase I trial evaluated the safety of giving SS1P as a continuous infusion in a similar patient population [86]. Although higher dosing was achieved, responses were not dramatically different from bolus dosing. Thus, the former dosing strategy was pursued going forward, due to the greater ease of administration. In both trials, pleuritis and CLS were observed as common toxicities, and no patients had an objective radiologic tumor response. In short and long infusion dosing strategies, 75% and 88% of patients developed neutralizing ADAs, respectively, after cycle 1. These ADAs were associated with the zero or near-zero peak plasma drug levels of SS1P. As cancer treatment typically requires sustained treatment for many cycles to have a successful anti-tumor effect, this presented a significant hurdle for the further clinical development of SS1P.

Combination therapies were next tested, using agents that could enhance efficacy, limit ADA formation, and mitigate toxicity of SS1P. A phase I trial for patients with untreated, surgically unresectable mesothelioma, combining SS1P with the standard of care chemotherapy agents pemetrexed and cisplatin, was undertaken. The response rate of the chemotherapy alone in a randomized trial performed in a similar population was previously found to be 41.3% [33]. With the addition of SS1P, 12 of 20 (60%) evaluable patients had a partial response (PR) and 3 had SD [87]. The cisplatin/pemetrexed regimen is strongly myelosuppressive. Despite this, 90% of patients treated on the SS1P combinations study developed ADAs by cycle 2, resulting in subtherapeutic maximum concentration (C_max_) beyond the first cycle. This data suggested that the addition of SS1P to chemotherapy for even a single treatment cycle might improve patient outcomes, however, additional interventions to minimize ADA development would be required for sustained treatment with SS1P.

It was found that selective lymphodepletion with the chemotherapy drugs pentostatin and cyclophosphamide (P+C) could combat ADA formation in mice immunized with iTox [90]. Subsequently, this regimen was advanced to clinical trials in patients with pre-treated, advanced mesothelioma [88]. In the first cycle, patients were pre-treated with an induction regimen of P+C for 9 days, followed by SS1P given on the standard schedule. A shorter three-day P+C induction was given in subsequent cycles. The primary objectives were to determine if SS1P with P+C was safe and effective in delaying anti-SS1P neutralizing antibody formation. Only 2 of 10 patients in the study developed ADAs after cycle 1. Interestingly, objective near complete responses were reported in three of eight patients in the study who received two or more cycles of therapy. Neither single agent pentostatin nor cyclophosphamide has activity against mesothelioma [91]. These responses were not only marked but durable, lasting at least 16 months after treatment. These unprecedented results in the mesothelioma population provide strong evidence that MSLN-targeted iToxs can be effective in treating solid tumors. 

### 4.2. LMB-100 for Mesothelioma Patients

LMB-100 was designed with the goal of increasing duration of therapy by designing a molecule that provoked less ADA formation. The new generation clinical lead was initially tested in a multicenter, international, single-arm, open-label phase 1 trial sponsored by Roche (NCT02317419). Eligible patients were required to have locally advanced or metastatic malignant solid tumors, known to express mesothelin and at least one prior systemic treatment for their disease. Roche terminated the trial before a MTD could be determined. The phase 1 evaluation was continued at the National Cancer Institute (NCI), under a separate clinical protocol (NCT02798536). 

For the NCI study, inclusion criteria were changed to include only participants with mesothelioma. Patients with sarcomatoid subtype or greater than 50% sarcomatoid component in their biphasic disease were excluded, as the sarcomatoid subtype does not express MSLN. Ten patients were treated with single agent LMB-100 on the NCI study [92]. It was recently reported that the MTD of 140 mcg/kg was determined for single agent LMB-100. Based upon patient derived mesothelioma xenograft models supporting the in vitro synergistic antitumor efficacy of LMB-100 and nab-paclitaxel, NCT02798536 was expanded to evaluate the MTD of LMB-100 in combination with nab-paclitaxel. A total of 11 patients were treated with the combination. Although the Roche and NCI studies have completed accrual, the results have not yet been published. 

Following treatment with LMB-100 on NCT02798536, a total of nine patients received the anti-PD1 antibody pembrolizumab as their next therapy. Durable objective tumor responses were seen in four of these patients, including one complete and three partial responses. All four of these patients were found to harbor tumors with positive PD-L1 expression. While the progression-free survival for the seven evaluable patients who received post-LMB-100 pembrolizumab was 8.7 months, the patient who experienced a complete response remained disease free after over 33 months following treatment [92]. This anecdotal clinical data have formed the basis for a phase II trial evaluating whether LMB-100 (140 mcg/kg), for two cycles followed by pembrolizumab for up to two years, is effective in treating patients with epithelioid or biphasic (with at least >50% epithelioid component) mesothelioma (NCT03644550), or with non-squamous non-small cell lung cancer (NCT04027946). Study accrual is ongoing.

In summary, single-agent LMB-100 is safe and generally well-tolerated at 140 mcg/kg in malignant mesothelioma. Its effectiveness as a single agent for this disease remains unproven. Multiple trials are currently ongoing, to evaluate whether synergistic, sequential, or combinatorial treatment regimens will prove beneficial to patients with malignant mesothelioma.

### 4.3. LMB-100 for Pancreatic Cancer Patients

MSLN is highly expressed in pancreatic ductal adenocarcinoma, the most common subtype of pancreatic cancer. The elevated expression of MSLN alone and co-expression with MSLN binding partner MUC-16/CA-125, has been associated with decreased progression-free and overall survival [3,47]. Given the pre-clinical data showing synergy with taxanes [74], a phase I/II clinical trial was conducted, evaluating the safety and efficacy of LMB-100 and nab-paclitaxel for patients with advanced pancreatic cancer (NCT02810418) [89]. The study found a >50% decrease in the pancreas cancer serum tumor marker CA 19-9 in 7 of 17 evaluable patients, along with a radiologic confirmed PR in one patient. Despite exciting evidence of clinical activity, severe toxicities related to CLS were seen in several patients, including cardiac edema. Furthermore, high titer ADA development occurred in nearly all patients before a third cycle of LMB-100 could be administered, suggesting that few patients would obtain further benefit beyond six weeks of treatment. This study again emphasized the need for identifying a safe regimen that could prevent or delay ADA development. 

## 5. Overcoming Challenges

### 5.1. Anti-Drug Antibodies

**Figure 2:**
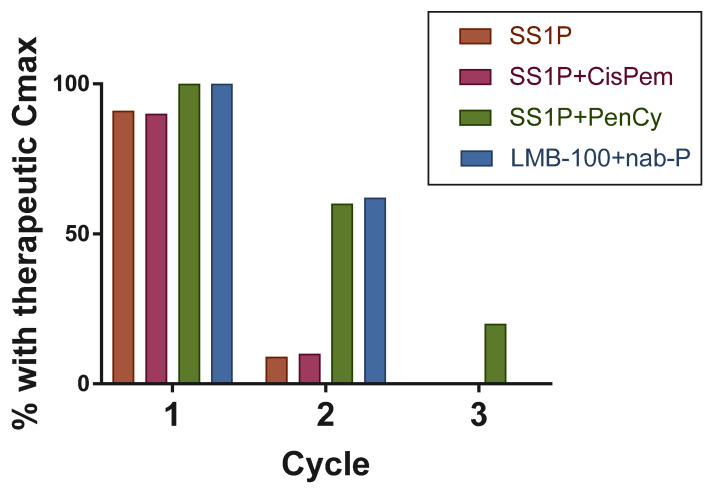
Rates of therapeutic drug levels by treatment cycle for each clinical trial of MSLN-targeted iToxs.

In patients with solid tumors, ADA formation limits the number of effective iTox treatment cycles that can be given. In contrast to patients with hematologic malignancies, those with solid tumors have immune systems that are relatively intact. Introducing a bacterial toxin-based molecule into these patients causes significant host immune response. Almost all patients are unable to achieve therapeutic C_max_ beyond one cycle of SS1P or two cycles of LMB-100 treatment [85,86,87,89]. Thus, the formation of neutralizing ADAs remains a significant barrier to the iTox treatment of solid tumor patients (Figure 2). 

As mentioned above, the lymphodepleting regimen of P+C followed by SS1P delayed ADA formation and extended the number of cycles, where SS1P reached bioactive circulating blood levels in patients [88]. Unfortunately, the toxicity associated with the P+C/ SS1P regimen was untenable. Specifically, the chemotherapy caused severe lymphocyte depletion that lasted three or more months post-therapy and precluded patient treatment with other types of immunotherapy. This significantly impacted the ability of progressing patients to identify a subsequent treatment. 

The second generation mesothelin-targeted iTox LMB-100 was specifically designed to be less immunogenic [89]. In the Phase I study evaluating the safety of LMB-100 with nab-paclitaxel in pancreatic adenocarcinoma, 62% of patients achieved therapeutic C_max_ on cycle 2, a marked improvement over single-agent SS1P. However, the study had to be amended to limit treatment to two cycles, since further treatment was associated with infusion-related reactions related to the development of ADA. Although LMB-100 has decreased immunogenicity compared to SS1P, the improvement has not been large enough to better clinical outcomes, and further research is needed.

Given that adequate drug levels are very rarely achieved beyond cycle 2, the combination of iTox with immune-modulating drugs remains an important strategy to mitigate ADA formation. Clinical studies testing iTox combinations with single agent oral cyclophosphamide [93], rituximab [94], or cyclosporin A [95] failed to show these drugs could delay or eliminate ADA formation. Recently, pre-clinical work done by Mazor et al. found that combination of LMB-100 with the SEL-110 rapamycin nano-particle induced immune tolerance to LMB-100 in mice [96]. This was the rationale for a phase I trial evaluating the safety of LMB-100 in combination with SEL-110 for advanced mesothelioma (NCT03436732). Unfortunately, as per clinicaltrials.gov, the unexpected severe toxicity of the combination regimen was observed in two of four patients accrued including occurrence of grade 5 pneumonitis and grade 4 pericardial effusion. The study was closed due to this safety signal. Other agents that have shown promise at delaying or eliminating ADAs in the pre-clinical setting include bortezomib [97], low-dose methotrexate [98], and tofacitinib [80,81]. Given the strong pre-clinical data, relatively benign toxicity profile, oral route of administration, and common use in rheumatologic conditions, tofacitinib was selected for further clinical investigation. A phase I trial to investigate safety and efficacy of tofacitinib in delaying ADAs to LMB-100 in advanced pancreatic cancer is currently underway (NCT04034238). 

Others have explored alternative non-PE payloads as a means to avoid immunogenicity in solid tumor patients. For instance, the repeated systemic administration of VB6-845, an anti-EpCAM Fab linked to de-Bouganin plant ribotoxin, to 10 solid tumor patients did not result in the significant formation of ADAs [99]. Pre-clinical studies optimizing other low immunogenicity ribotoxins and RNase payloads, some of which are human-derived, are ongoing [100,101]. These payloads have not yet been utilized to target MSLN.

### 5.2. Toxicity

In addition to ADA formation, the toxicity of SS1P and LMB-100 presents an obstacle to clinical use. The dose-limiting toxicity (DLT) of SS1P is pleuritis. This adverse drug effect was attributed to on-target, off-tumor toxicity, resulting from the SS1P targeting of MSLN expressed on the pleural surface [85]. While pleuritis was so severe and common with SS1P treatment that high dose steroids were prophylactically administered to every patient with each dose of iTox, it has been rarely observed with LMB-100, even in the absence of steroid prophylaxis. Conversely, pericarditis was not observed with SS1P, but several cases of this probable on-target off-tumor cardiac toxicity have been observed in patients receiving LMB-100 combinations. Interestingly, serositis has not been a significant toxicity of mesothelin-targeted ADCs or vaccine therapies [102,103,104,105]. In the absence of pre-clinical data, this leads one to speculate that the mesothelial cells which form serosal tissues are differentially sensitive to varying payloads and/or have an immune privilege to avoid an anti-MSLN immune response induced by vaccine or drug attack. Differential payload sensitivity has been clearly observed amongst therapeutics targeting Her2. For instance, the Her2- targeted ADC ado-trastuzumab emtansine is a relatively safe FDA approved therapeutic, while a PE38-based immunotoxin targeting Her2 caused fatal on-target off-tumor hepatic toxicity during Phase 1 evaluation [106]. In addition, it has been observed that many cancer cell lines have differing sensitivities to PE38 versus PE24-based iTox, and it may be that mesothelial cells behave similarly. Alternatively, the delivery of varying therapeutics to the mesothelial surfaces may vary. SS1P has a significantly longer half-life than LMB-100, and is smaller than ADCs; these are both factors which could increase exposure of pleural mesothelial cells. Of course, this does not explain the diametrically different rates of pleuritis versus pericarditis or peritonitis seen with this molecule. Because the MSLN-binding domains of current iToxs and other MSLN-directed therapies do not recognize the murine isoform of MSLN, serositis has not been examined in the pre-clinical setting. A better understanding of the factors which contribute to serositis could result in the engineering of new molecules that cause less of this toxicity, a possible means of improving the iTox therapeutic window. 

**Figure 3:**
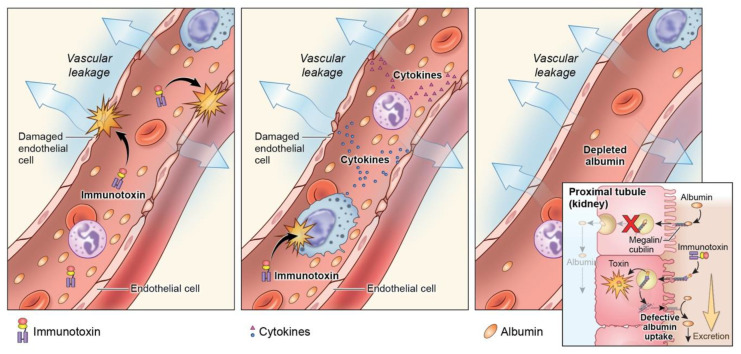
Proposed mechanisms of capillary leak syndrome (CLS). The left panel shows direct damage to endothelial cells by iTox. An alternate theory is that stimulation of immune cells by iTox induces release of a circulating factor which causes endothelial damage and CLS (middle panel). The right panel illustrates the hypothesis that iTox-induced loss of albumin in the proximal convoluted tubule of the kidney leads to hypoalbuminemia and CLS results from decreased osmotic pressure and/or compensatory renal signaling mechanisms.

CLS, a well-described toxicity associated with iTox treatment regardless of the target, remains problematic for MSLN-targeted iTox in the clinical setting [107]. CLS is frequently observed in patients receiving SS1P [85] and is the DLT of LMB-100 [89]. In addition, combination with nab-paclitaxel in pancreas cancer patients further augmented the rates and severity of CLS, despite ~30% dose reduction of iTox from the single agent MTD [89]. The precise mechanism of CLS has yet to be elucidated, despite considerable inquiry (Figure 3). 

Historically, iTox-mediated CLS was thought to be caused by direct damage to endothelial cells, through either off-target uptake, or the presence of specific binding domains within PE that could direct the toxin to endothelial cells [108]. There is currently no clinical evidence to support or refute this mechanism. It has also been hypothesized that systemic inflammation induced by the toxin moeity results in the secretion of a yet unidentified cytokine that induces CLS. Consistent with this hypothesis, the systemic inflammatory marker C-reactive protein does increase in patients following LMB-100 administration, but association between CLS and a specific cytokine has not been established [93]. More recently, Liu et al. have suggested that CLS may result from direct toxicity to proximal tubule cells in the kidney, causing a loss of albumin in the urine [109]. This loss of albumin reduces osmotic pressure in the vasculature, and favors the accumulation of fluid in the tissue at the expense of circulating volume, the clinical scenario that is observed in patients with CLS. This study further demonstrated that co-administration of l-lysine for renal protection can reduce renal damage in mice. This strategy has yet to be tested in patients.

### 5.3. Delivery

Large molecule therapeutics like iTox frequently face challenges with delivery to, and penetration within, solid tumors and is particularly problematic in a high stroma tumor like PDAC. The successful tumor delivery of mesothelin-targeted imaging agents utilizing full monoclonal antibodies has been reported in patients with mesothelioma, ovarian cancer and PDAC, providing proof that MSLN-targeting can result in tumor-specific delivery [110,111]. The efficiency of SS1P and LMB-100 delivery to patient tumors in the clinical setting has not been investigated. Methods for direct detection of these protein therapeutics in patient tissue are insensitive. Even if direct detection were possible, it would remain difficult to reliably discriminate whether uptake into cancer cells has occurred, or whether the iTox has been sequestered in the tumor microenvironment, where it has no activity. Unfortunately, a robust biomarker for iTox protein synthesis activity in patient tumor tissue has not been identified, despite a broad analysis examining changes in tumor-associated factors following iTox treatment [112]. Studies in mouse xenograft tumors suggest that iTox delivery is heterogenous [113], and can be improved by co-administration of a stromal-cell killing chemotherapy such as taxane [114]. The combination of iTox with targeted stromal modifying agents has not yet been pursued. 

The use of novel antibody formats also has the potential to improve delivery and internalization. Nanobodies, naturally occurring single domain antibodies frequently derived from camelids or sharks, may penetrate solid tumors more effectively, due to their smaller size. This format has recently been utilized to make anti-Her2 [115] and anti-glypican-3 iTox [116] that appear to have improved pre-clinical activity, and it will be interesting to follow the clinical development of these molecules. Going to larger antibody format may also have benefits. Recently, a trimerbody immunotoxin targeting the colorectal cancer-associated tumor marker carcinoembryonic antigen (CEA), IMTXCEAαS, was described [117]. The multi-valent format was reported to have increased activity compared to the monomeric form in mice bearing human tumor xenografts. It is unclear how well this format would translate to MSLN-targeting, as any gains in anti-tumor activity may be non-productively counterbalanced by increased toxicity to MSLN-expressing serosal membranes. 

An additional concern for delivery is the consequences of physiologic shedding of MSLN from the cancer cell surface. Because iToxs require tumor cell internalization for anti-tumor activity, there is no bystander effect. Therefore, MSLN that accumulates in the tumor extracellular fluid after shedding could potentially act as a decoy receptor that limits iTox activity. While mathematical modeling suggested that the shedding of MSLN within the tumor microenvironment could improve delivery by storing a depot of iTox [118], experimental modeling reinforced concerns that high concentrations of shed MSLN within the tumor microenvironment acts as a site barrier to MSLN reaching the tumor cell surface [119]. Furthermore, when iTox reaches the cell surface, if internalization of surface MSLN-iTox complex is slower than the rate of MSLN shedding, this could also limit the successful accumulation of toxin within the tumor cell cytoplasm. In fact, pre-clinical data demonstrated that tumor cells expressing mutant MSLN constructs with impaired shedding are more sensitive to iTox [120]. A further understanding of the MSLN shedding process and its effect on the delivery of iTox to tumor cells may enable clinical co-interventions that maximize the delivery of MSLN-targeted iTox to cancer cells. 

## 6. Summary

MSLN-targeted iToxs have shown great promise in both the pre-clinical and clinical settings for solid tumors like mesothelioma and pancreatic cancer that have limited treatment options. Substantial progress has been made since the first use of MSLN as a target for this unique class of therapeutics. Nevertheless, significant challenges remain, and much work still needs to be done in the field. Future directions of research will likely focus on mitigating ADA formation, minimizing CLS, and seeking drug combinations that enhance the anti-tumor effect. 

## Figures and Tables

**Table 1:** Summary of clinical trials of MSLN-targeted immunotoxins. Trials without references do not have published results at the time of submission of this review.

iTox	NCT	Trial Name	Location	Status	No. of Patients	ORR *	Ref
SS1P	00066651	Phase I Study of SS1(dsFv)-PE38 Anti-Mesothelin Immunotoxin in Advanced Malignancies: I.V. Infusion QOD Dosing	NCI	Closed	*n* = 34 20 mesothelioma 12 ovarian cancer 2 PDAC	4/33 ^	[85]
SS1P	00006981	Phase I Study of SS1(dsFv)-PE38 Anti-Mesothelin Immunotoxin in Advanced Malignancies: Continuous Infusion × 10 Days	NCI	Closed	*n* = 24 16 mesothelioma 7 ovarian 1 PDAC	1/24	[86]
SS1P	01445392	A Phase I, Single Center, Dose-Escalation Study of SS1(dsFv)PE38 Administered Concurrently with Pemetrexed and Cisplatin in Subjects with Unresectable Malignant Epithelial Pleural Mesothelioma	NCI	Closed	*n* = 24 mesothelioma	12/20	[87]
SS1P	01362790	A Pilot/Phase 2 Study of Pentostatin Plus Cyclophosphamide Immune Depletion to Decrease Immunogenicity of SS1P in Patients with Mesothelioma, Lung Cancer or Pancreatic Cancer	NCI	Closed	*n* = 11 mesothelioma	3/10	[88]
LMB-100	02317419	Phase IA/IB, Open-Label, Multicenter, Multiple Ascending Dose Study Followed by an Extension Phase to Evaluate the Safety, Tolerability, Pharmacokinetics and Activity of R06927005, An Anti-Mesothelin (MSLN) Recombinant Cytolytic Fusion Protein (cFP), Administered Either Alone (Part A) or in Combination with Gemcitabine and Nab-Paclitaxel (Part B) in Patients with Mesothelin-Positive Metastatic and/or Locally Advanced Malignant Solid Tumors	Multicenter/multi-national: USA (NCI) Canada, Denmark France	Terminated	*n* = 15 7 mesothelioma 3 ovarian 3 PDAC 2 gastric	NR	-
LMB-100	02798536	A Phase I Study of the Mesothelin-Targeted Immunotoxin LMB-100 With or Without Nab-Paclitaxel in Patients with Malignant Mesothelioma	NCI	Closed	*n =* 10 mesothelioma	NR	-
LMB-100	03436732	A Phase I Study of the Mesothelin-Targeted Immunotoxin LMB-100 in Combination with SEL-110 in Subjects with Malignant Pleural of Peritoneal Mesothelioma	NCI	Terminated	*n =* 5 mesothelioma	NR	-
LMB-100	02810418	A Phase I/II Study of Mesothelin-Targeted Immunotoxin LMB-100 Alone or in Combination with Nab-Paclitaxel in People with Previously Treated Metastatic and/or Locally Advanced Pancreatic Ductal Adenocarcinoma and Mesothelin Expressing Solid Tumors	NCI	Closed	*n =* 40 37 PDAC 1 rectal cancer 1 mesothelioma 1 ampullary	1/40	[89]
LMB-100	04034238	A Phase I Study of Mesothelin-Targeted Immunotoxin LMB-100 in Combination with Tofacitinib in Persons with Previously Treated Pancreatic Adenocarcinoma, Cholangiocarcinoma and Other Mesothelin Expressing Solid Tumors	NCI	Recruiting	Up to 45 planned	NR	-
LMB-100	03644550	Phase II Study of the Anti-Mesothelin Immunotoxin LMB-100 Followed by Pembrolizumab in Malignant Mesothelioma	NCI	Recruiting	Up to 100 planned	NR	-
LMB-100	04027946	A Phase II Study of LMB-100 Followed by Pembrolizumab in the Treatment of Adults with Mesothelin-Expressing Non-Squamous Non-Small Cell Lung Cancer	NCI	Recruiting	Up to 38 planned	NR	-

NCI = National Cancer Institute; ORR = objective response rate; NR = not reported; * Partial response unless otherwise indicated, of evaluable patient denominator; ^ Minor responses, defined by decreased tumor area ≥20% but <50% from baseline, and lasting ≥4 weeks.
